# Impact of International Tourists’ Co-creation Experience on Brand Trust, Brand Passion, and Brand Evangelism

**DOI:** 10.3389/fpsyg.2022.866362

**Published:** 2022-03-24

**Authors:** Gustave Florentin Nkoulou Mvondo, Fengjie Jing, Khalid Hussain, Shan Jin, Muhammad Ali Raza

**Affiliations:** ^1^School of Business, East China University of Science and Technology, Shanghai, China; ^2^Department of Management Sciences, COMSATS University Islamabad, Sahiwal, Pakistan; ^3^Department of Business Administration, National College of Business Administration and Economics (NCBA&E), Multan, Pakistan

**Keywords:** co-creation experience, brand evangelism, brand trust, brand passion, international tourists, theory of engagement

## Abstract

Drawing on the theory of engagement, the present study aims to examine the outcomes of the co-creation experience in a realistic co-creation setting, a hotpot restaurant. To this end, the current research links the relationship marketing literature to hospitality and tourism research and formulates a novel framework by incorporating tourists’ co-creation experience, brand evangelism, brand trust, and brand passion in an integrated conceptual model. Using a quantitative research design, a total of 453 international tourists were surveyed in China. The findings revealed that co-creation experience dimensions positively impact brand evangelism, trust, and passion. Additionally, we found that brand trust and brand passion positively affect brand evangelism. We also confirmed the mediating effect of brand trust and brand passion in bridging the co-creation experience and brand evangelism. This study offers valuable insights for restaurant brand managers regarding attracting and engaging foreign travelers with their service businesses.

## Introduction

China has emerged as a top destination for global tourists since the Open Door Policy was enacted in 1978. The period from 2001 to 2019 witnessed an incredible increase in the number of visitors, which rose from 89.1 to 145 million, accounting for an overall growth rate of about 63% (Statista, 2021). Tourists travel for different motives, but visiting local restaurants constitutes an integral part of their stay (Hussain et al., 2018). According to Liu and Jang (2009), Chinese cuisines are exceedingly favored among tourists owing to their high diversification and unique taste. This popularity has led to intense competition among restaurants brands; however, they face many challenges in engaging tourists with their brand, leading to an uncertain future. Their primary objective has been to generate profit rather than understand customers’ needs and expectations and offer value to them. The old-fashioned good-centered dominant logic in which customers are passive receivers of value has shown its inefficacy in retaining customers and engaging them long-term. Accordingly, the customers’ role has shifted from passive receivers of value to co-creators of values alongside the organization (Vargo and Lusch, 2004); therefore, restaurant brands should consider tourists as part of stakeholders and involve them in the value co-creation activity.

Since conception, value co-creation has become a very influential concept, as practitioners and scholars have increased their interest in service-dominant logic (Vargo and Lusch, 2004). Service-dominant logic implies that customers are always co-creators of value. Customers involved in value co-creation feel less deprived and more fulfilled (Navarro et al., 2015). Therefore, co-creation experience (CCE) has received substantial research attention in tourism marketing (Verhoef et al., 2010; Shaw et al., 2011), as an increasing number of firms involve customers in the co-creation process (Jaakkola et al., 2015; Zhang et al., 2016). CCE is defined as the benefits customers expect from the co-creation activity (Verleye, 2015). Extant literature has linked CCE with customer satisfaction (Alarcón López et al., 2017; Hussain et al., 2020), purchase intention (Blasco-Arcas et al., 2014), customer loyalty (Mathis et al., 2016), revisit intention (Hurriyati and Sofwan, 2015; Meng and Cui, 2020) and memorability (Campos et al., 2016; Rachão et al., 2019).

Nonetheless, CCE is limited in three important perspectives. First, while scholars have investigated CCE in the hospitality and tourism context (i.e., Tu et al., 2018; Wu et al., 2018), research on the effect of CCE on tourists’ dining experience remains scarce. Grönroos (2011) revealed that cooking a meal at a restaurant is a comprehensive example of the value co-creation process. Additionally, recent research has confirmed that the restaurant industry provides a platform to investigate customer experience in a co-creation setting (Hussain et al., 2019, 2020). For instance, restaurants, such as McDonald’s and Inamo, offer customers the opportunity to co-create by selecting food through a touchscreen. Chinese hotpot restaurants offer customers a complete CCE by involving them in the cooking process (Hussain et al., 2020). Second, although researchers have acknowledged the importance of CCE, empirical work on its outcomes is still limited (Verleye, 2015). Scholars have primarily paid attention to the antecedent of CCE (e.g., Cova et al., 2011). Third, the economic (e.g., sales growth) and customer benefits of CCE (e.g., customer retention and customer acquisition) are poorly known. An in-depth analysis of CCE literature reveals a scarcity of research investigating the CCE-economic and customer benefit link. The purpose of the current study is to address these mentioned gaps in the literature of CCE. Therefore, the ultimate question is: What is the substantial return on marketing investment outcomes for firms promoting the CCE in the hospitality context?

We propose that brand evangelism (BE), a high level of positive word-of-mouth communication, is a possible outcome for firms. Matzler et al. (2007) defined BE as a more active and committed way of spreading positive opinions and trying fervently to convince or persuade others to get engaged with the same brand. According to Doss (2014), the “evangelist” is an unpaid brand representative who evangelizes others on behalf of the brand. Samson (2006) stated that customer responses to evangelism are more substantial and effective than word-of-mouth, which is the outcome of positive consumer involvement. Although anecdotal proof affirms the importance of CCE, the influence of CCE on BE is largely unresearched.

Consumer–brand relationships have attracted considerable attention among scholars and brand managers as they can influence consumer behavior and marketplace advantages for firms (Albert and Merunka, 2013; Fetscherin and Heinrich, 2015). However, researchers have overlooked the relationship between CCE and consumer–brand relationships, including brand trust (BT) and brand passion (BP). According to Chaudhuri and Holbrook (2001), BT refers to the willingness of the average consumer to rely on the ability of the brand to provide its stated function. Consumers’ trust in brands is valuable to brands in both online and offline markets (Becerra and Korgaonkar, 2011). As a driving force for consumer–brand relationship, Sternberg (1986) explained that passion is a “hot” element, resulting in romance, physical attraction, adoration, and idealization of a significant other. According to Bauer et al. (2007), the growing relevance of passionate brands in marketing requires examining BP determinants. Based on the theory of engagement, The present study investigates the effect of international tourists’ CCE on BT, BP, and BE in a realistic and routinely performed co-creation setting, a hotpot restaurant.

The current research makes several contributions to the fields of branding, hospitality, and tourism, primarily through the discussion of two critical concepts: the CCE of customers and BE. Mathis et al. (2016) emphasized that CCE can influence brand outcomes. To the best of our knowledge, this study is the first attempt to offer an integrated model of CCE by investigating its effect on BE in the hospitality and tourism context. It identifies how CCE enhances BT and BP and stimulates BE. Hussain et al. (2020) explained that CCE benefits both customers and restaurant brands.

study provides a framework for restaurant brand managers regarding optimizing their service and enhancing their business through the retention and acquisition of valuable customers.

## Literature Review and Hypotheses Development

### Co-creation Experience

The value co-creation process comprises various actors, including the beneficiary, who decides the value, while other actors contribute to developing and presenting value proposals (Vargo and Lusch, 2004). Ahn et al. (2019) outlined value co-creation behavior as consumers cooperating with other actors to engage in a series of important value-creation activities. In contrast, CCE refers to the mental state resulting from the customers’ participation in the value co-creation activity (Chen, 2018).

Several scholars have examined the concept of co-creation in the tourism (Hurriyati and Sofwan, 2015; Rachão et al., 2019; Mohammadi et al., 2020) and hospitality sectors (Kallmuenzer et al., 2019; Gibbs et al., 2021), as co-creation complements tourism while providing tourists with the opportunity to participate in services. Ramaswamy and Ozcan (2018) posited that co-creation facilitates interactional creation across interactive system environments (afforded by interactive platforms), entailing agency engagements and structuring organizations. According to Jaakkola et al. (2015), CCE is multidimensional as both a phenomenon and a concept. Merz et al. (2018) highlighted its multidimensional nature using two higher-order factors: customer-owned resources and customer motivation. Verleye (2015) highlighted four dimensions of CCE, namely, hedonic experience (HE), cognitive experience (CoE), social experience (SE), and economic experience (EE).

According to Zhang et al. (2020), hedonic reaction is the positive emotional state resulting in customer satisfaction; HE is seen as the pleasurable experience customers expect from the CCE. Customers participating in the value co-creation process seek fun, pleasure, and entertainment regardless of extrinsic rewards (Chen, 2018). CoE is knowledge about service, products, and technologies that customers expect to gain from a co-creation activity (Verleye, 2015). It helps customers to explore news ways to use products and learn from other participants’ co-creation efforts. Füller (2010) stated that customers seeking CoE are intrinsically motived and stimulated by their desire to generate and implement creative ideas for their own sake. At Chinese hotpot restaurants, participation in value co-creation offers customers the opportunity to keep pace with new ideas and hone their skills and ability to perform a specified task. SE is the benefit of connecting with like-minded people during the co-creation activity. Customers expect to gain better status and social esteem from their value co-creation process. According to Füller (2010), this experience builds on the desire of an individual for social identity, recognition, and development of skills that improve communication with the outside world. While some customers participate in the co-creation activity to hone their skills and meet like-minded people, others expect pragmatic and economic benefits. EE refers to the reduction of risks associated with receiving inappropriate products or services and the compensation in line with the effort made (Verleye, 2015). Verleye (2015) found that all four dimensions positively impact the overall co-creation experience (OCCE).

The scale developed by Verleye (2015) presents higher construct validity, reliability, and internal consistency. She stressed that further research should validate the scale in different co-creation situations; therefore, we utilized the exact dimensions to investigate the relationship between CCE, BT, BP, and BE in a restaurant setting. Recent studies have endorsed Verleye’s dimensions (e.g., Hussain et al., 2020).

### Brand Evangelism

Organizations face several challenges because of a highly connected marketplace; they are keen to focus on the smaller but most influential customer category known as brand evangelists (Marticotte et al., 2016). The reason for this focus is their potential ability to actively disseminate brand-related experiences among others, embrace a particular brand intensively, persuade others to experience the brand, and dissuade others from buying rival brands (McConnell and Huba, 2003). Many studies argue that positive word-of-mouth or assertive support behavior, such as brand advocacy and brand defense, is derived from the consumer–brand relationship (Hsu, 2018). Mishra et al. (2021) found that customer–brand relational constructs, including brand trust, brand affect, and brand identification, strongly impact BE in terms of purchase intention and brand referral behavior. Harrigan et al. (2020) highlighted that value co-creation leads to brand advocacy and brand defense, which are intrinsically related to BE.

Sharma et al. (2021) outlined that brand community engagement is related to BE, and BE mediates the relationship between brand community engagement, brand defense, and brand resilience. Matzler et al. (2007) argued that a focus on word-of-mouth communication alone does not reveal customers’ behavior and their power to convince others about the brands they like; therefore, BE has emerged as an important concept in the consumer–brand relationship literature. Samson (2006) posited that BE generates a greater and more effective customer response than word-of-mouth. BE has facilitated small brands and benefited large organizations, including Southwest Airlines, IBM, and Build-A-Bear Workshop (McConnell and Huba, 2003).

According to Badrinarayanan and Becerra (2013), BE is customers’ behavioral support for a particular brand, including purchasing a specific brand, convincing others about it, and recommending it to others. Our study adopted the multidimensional approach of BE developed by Badrinarayanan and Becerra (2013). They found that BE is based on three dimensions: brand purchase intention (BPI), positive brand referrals (PBR), and oppositional brand referrals (OBR). Fishbein et al. (1977) defined BPI as a subjective inclination toward a brand or product. Therefore, it is a measure of the strength of consumer intention to decide to purchase a brand in the future (Yeo et al., 2020). Brand evangelists, considered active supporters of a brand, show behavioral support by purchasing products of that brand. PBR can be defined as the brand evangelists’ active behavioral support for a brand, which they show by disseminating favorable opinions, recommending it to others, and attempting to convince others to engage with the same brand. OBR is a negative attitude toward competing brands, resulting from attachment and loyalty to a brand. The strategies employed by brand evangelists go beyond purchasing the brand to include denigrating rival brands that present a threat (Doss, 2014). According to Badrinarayanan and Becerra (2013), research on the antecedents of BE is still limited, so there is a need to broaden the scope for a better understanding of what leads to evangelistic behaviors.

### Brand Trust

BT plays a vital role in relational exchange (Riorini and Widayat, 2015; Ling et al., 2021). According to Chaudhuri and Holbrook (2001), the foundation of BT relies on the brand’s ability to perform its stated function; therefore, the reliability of a brand is the root of BT (Ballester and Munuera-Alemán, 2005; Villagra et al., 2021). Trust has become a very influential concept in branding (Husain et al., 2022). Morgan and Hunt (1994) stated that trust exists when the concerned parties have confidence in each other’s integrity and reliability. In the tourism and hospitality sector, Kim et al. (2014) suggested that a well-renowned celebrity is seen as reliable and can be influential in attracting customers’ attention and highlighting a positive image for hotels.

BT has strong positive relations with brand love (Albert and Valette-Florence, 2010) and BP (Gilal et al., 2020). Fehr and Anne (1988) stated that trust relates to affection more than passion in terms of interpersonal relations. However, similar to passion, BT encompasses both cognitive and affective dimensions (Ling et al., 2021). Baumann and Le Meunier-FitzHugh (2013) reported that trust among parties emerges from internally guaranteed certainty, which leads to brand value co-creation and ensures the other party will not act opportunistically. Their study further highlighted that consumers’ BT is a core element that makes consumers brand evangelists.

Several scholars have investigated the relationship between customer experience and BT (Ha and Perks, 2005; Walter et al., 2013; Guan et al., 2021); however, they have largely overlooked the relationship between CCE and BT even though both concepts are fundamental in measuring consumer behavior.

### Brand Passion

Many scholars have investigated the concept of passion in marketing. Bauer et al. (2007) stated that brand uniqueness is one of the principal drivers of consumers’ BP from a theoretical perspective. According to Matzler et al. (2007), individual factors, such as consumer personality, influence BP. Their findings contradicted those of Bauer et al. (2007), who found that the extroversion trait in consumer personality is highly effective for fostering BP.

Gilal et al. (2018) stressed that passion plays a crucial role in shaping positive word-of-mouth communications. Moreover, passion improves consumers’ desire to pay for a premium brand (Swimberghe et al., 2014; Gilal et al., 2018), consumer brand loyalty (Hemsley-Brown et al., 2016), brand advocacy, brand community engagement, social media support, and purchase intention (Pourazad and Pare, 2015; Mukherjee, 2020). Keh et al. (2007) posited that BP represents the zeal and enthusiasm features of the consumer–brand relationship. Matzler et al. (2007) concluded that when consumers are passionate about a brand, they develop a deep emotional relationship with it and may miss it or feel lost if the brand is unavailable. Therefore, BP is intrinsically related to consumer behavior (D'lima, 2018; Gilal et al., 2021a). Thomson et al. (2005) highlighted that BP emerges from an intense and aroused positive feeling toward a brand. Similar to BT, the literature has overlooked the relationship between CCE and BP. Therefore, this study attempts to address this gap in the literature.

### Hypotheses Development and Theoretical Framework

#### Customers’ Co-creation Experience and Brand Evangelism

The constructs of CCE and participation in value co-creation are intrinsically related. CCE is derived from the experience (or benefits) that customers gain from participating in the value co-creation activity.

In the value co-creation process, values are co-created by customers, for customers through experiencing the service and sharing them with others in the user community. Harrigan et al. (2020) outlined that value co-creation is positively related to BE, and Javed et al. (2015) posited that customers are more likely to spread positive opinions and defend what they helped create. Seifert and Kwon (2019) stated that co-creation efforts generate customer recommendations. They further posited that Yi and Gong (2013) use the term customer citizenship behavior to describe evangelistic behaviors, which, according to Badrinarayanan and Becerra (2013), comprise BPI, PBR, and OBR.

According to the theory of engagement, a positive customer experience with a brand leads to customer engagement, which comprises direct contributions (brand purchase) and indirect contributions (incentivized referrals and social media conservations; Pansari and Kumar, 2017). Navarro et al. (2015) stressed that customers involved in value co-creation feel less deprived and more fulfilled. Accordingly, customers participating in the value co-creation process are more likely to have a good experience with the firm, which will eventually stimulate brand purchase intention and positive word-of-mouth messages. Therefore, we hypothesize that:

*H1*: CCE dimensions of (a) hedonic, (b) cognitive, (c) social, (d) economic, and (e) overall co-creation experience positively influence BE dimensions of brand purchase intention, positive brand referrals, and oppositional brand referrals.

#### Customers’ Co-creation Experience and Brand Trust

CCE is the customer experience resulting from the co-creation activity. Guan et al. (2021) investigated the relationship between customer experience and BT and found that experience is related to trust, which is consistent with the findings of Ha and Perks (2005) and Walter et al. (2013). Kamboj et al. (2018) found that customer participation, which is related to CCE, positively impacts BT.

Several scholars have examined the relationship between BT and customer satisfaction. They found that BT and customer satisfaction are positively related (Liao et al., 2011; Umar and Bahrun, 2017; Diputra and Yasa, 2021). In their theory of engagement, Pansari and Kumar (2017) posited that a positive/negative customer experience affects the level of satisfaction. Hussain et al. (2020) found that CCE impacts customer satisfaction. Based on the above discussion, we hypothesize that:

*H2*: CCE dimensions of (a) hedonic, (b) cognitive, (c) social, (d) economic, and (e) overall co-creation experience positively influence BT.

#### Customers’ Co-creation Experience and Brand Passion

In an increasingly competitive and connected marketplace wherein firms fulfill customers’ needs and expectations, marketing scholars and practitioners have stressed that building a highly emotional consumer–brand relationship is vital in consumer marketing (Huber et al., 2015). Scholars have found that passion and emotion are intrinsically related. According to D'lima (2018), emotion is a dimension of brand passion; therefore, passion is a strong or intense emotion. Bauer et al. (2007) stated that BP is the emotional attachment to a brand and Matzler et al. (2007) posited that passion engages emotional relationships.

According to Hussain et al. (2020), CCE is related to emotional brand attachment. Moreover, Pansari and Kumar (2017) posited that a positive/negative customer experience affects the level of emotions customers have for a firm (Theory of engagement). Accordingly, we hypothesize that:

*H3*: CCE dimensions of (a) hedonic, (b) cognitive, (c) social, (d) economic, and (e) overall co-creation experience positively influence BP.

#### Brand Trust and Brand Evangelism

BE is considered a high-level of word-of-mouth communication. Previous research found that BT is related to service outcomes, such as word-of-mouth communication (Wang et al., 2018) and purchase intention (Chae et al., 2020). Additionally. Scholars have investigated the relationship between BT and BE, finding that trust positively stimulates evangelistic behaviors (Badrinarayanan and Becerra, 2013; Doss, 2014; Riorini and Widayat, 2015). Therefore, we hypothesize that:

*H4*: BT positively influences BE dimensions of brand purchase intention, positive brand referrals, and oppositional brand referrals.

While it is customary to refer to customers as “the audience,” implying a more passive role, Oetting and Jacob (2007) posited that firms should involve customers in the co-creation process by offering them a more active role. BT is achieved through mutual experience and activities and is an important construct for a successful relationship between firms and customers.

According to Pansari and Kumar's (2017) theory of engagement, customer experience can lead to customer satisfaction which, in turn, leads to customer engagement. They posited that customer engagement comprises direct contributions (brand purchase) and indirect contributions (incentivized referrals and social media conservations). Badrinarayanan and Becerra (2013) outlined that BE dimensions comprise brand purchase (i.e., BPI) and referrals (i.e., PBR and OBR). Walter et al. (2013) found that customer experience is related to BT, and Diputra and Yasa (2021) found that BT impacts customer satisfaction.

Based on the above discussion, we posit that CCE leads to BT, which in turn, leads to brand evangelistic behavior. Therefore, we hypothesize that:

*H5*: BT mediates the relationship between the CCE dimensions of (a) hedonic, (b) cognitive, (c) social, (d) economic, and (e) overall co-creation experience, and BE dimensions of brand purchase intention, positive brand referrals, and oppositional brand referrals.

#### Brand Passion and Brand Evangelism

Marketing scholars have highlighted that BP has certain causes and leads to certain outcomes, both of which contribute to understanding consumer behavior (D'lima, 2018). Bauer et al. (2007) showed that BP has a positive impact on BPI and positive word-of-mouth. Gilal et al. (2021b) found that BP influences purchase intention, and Ghorbanzadeh et al. (2020) found that BP is related to positive word-of-mouth intention. Matzler et al. (2007) found that BP leads to BE. Accordingly, we hypothesize that:

*H6*: BP positively influences BE dimensions of brand purchase intention, positive brand referrals, and oppositional brand referrals.

**Figure 1:**
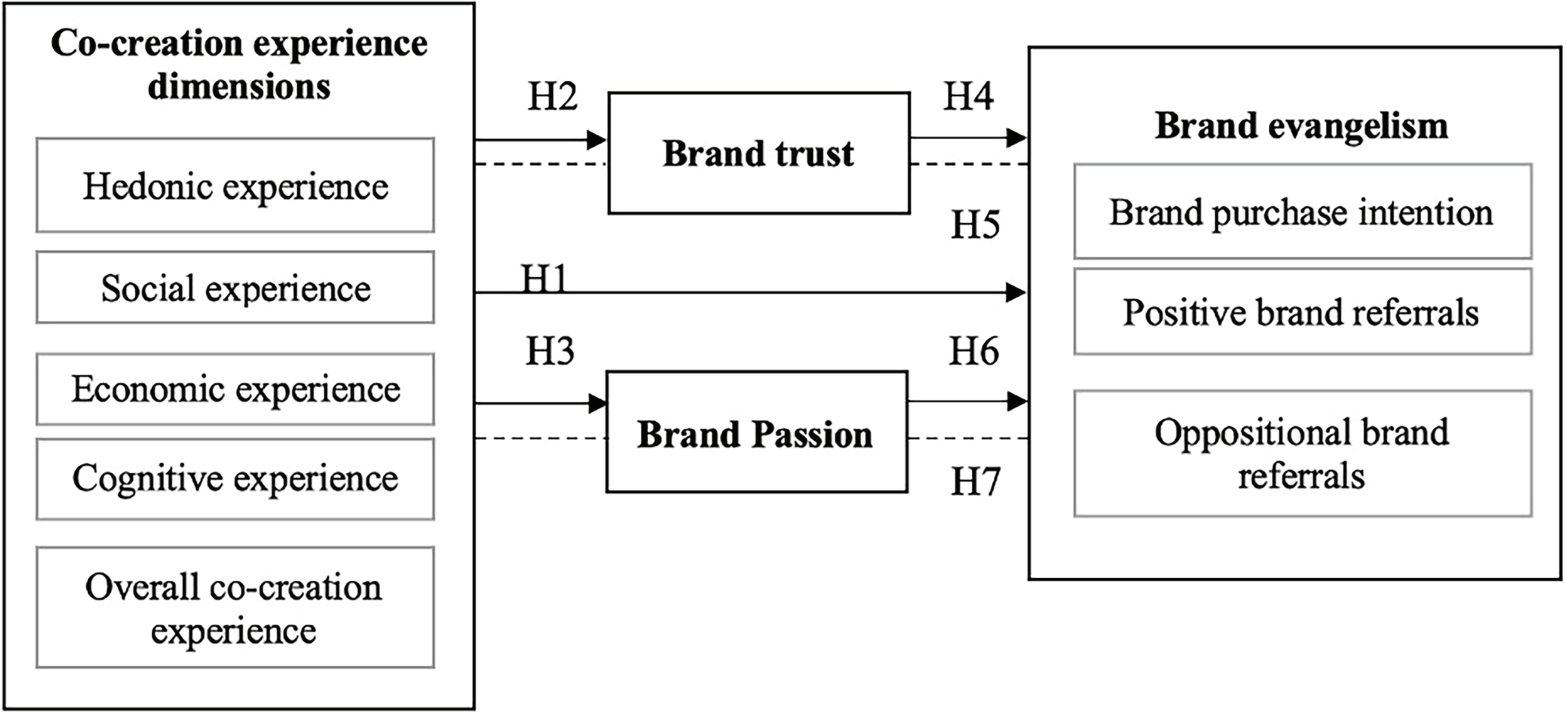
Theoretical framework.

According to Bauer et al. (2007), BP is the emotional attachment to a brand. Pansari and Kumar (2017) posited that customer experience is likely to lead to emotions which in turn leads to customer engagement. They further asserted that customers showing positive emotions would assist firms with behaviors, such as brand advocacy. Pourazad et al. (2019) found that BP is related to brand advocacy. Accordingly, we hypothesize that:

*H7*: BP mediates the relationship between CCE dimensions of (a) hedonic, (b) cognitive, (c) social, (d) economic, and (e) overall co-creation experience, and BE dimensions of brand purchase intention, positive brand referrals, and oppositional brand referrals (Figure 1).

## Methodology

### Measurement Instruments

The items and instruments used in the questionnaire to measure the constructs were adapted from previously validated studies to maintain reliability and validity. We used twenty-three items to assess CCE: six items for EE, three items for HE, five items for SE, five items for CoE, and four items for OCCE. All items were adapted from Verleye (2015). We measured the BE dimensions with ten items: four items for BPI, three items for PBR, and three items for OBR, adapted from Badrinarayanan and Becerra (2013). We measured BT using five items adapted from Chaudhuri and Holbrook (2001) and Munuera-Alemán et al. (2003), whereas for BP, we used five items adapted from Mostafa and Kasamani (2020) and Thomson et al. (2005). all the items were slightly modified from the existing literature to suit the study context. For example, the original item “I spread positive word of mouth about the brand” was adapted to “I spread positive word of mouth about my favorite hotpot brand.” Two marketing professors reviewed the questionnaire to ensure face and content validity; we distributed the questionnaire after applying their suggestions. Moreover, to measure all the items, we used a 5-point Likert scale, ranging from 1 = *strongly disagree* to 5 = *strongly agree*.

### Selection of the Co-creation Setting

In 2017, there were approximately 300,000 hotpot restaurants across China, and in 2020, 13,000 new restaurants were opened to meet increasing demand (Statista, 2020). Chinese hotpot restaurants provide customers with a complete CCE as customers can cook their own food. These restaurants provide customers with raw food and ingredients, which they mix up and cook according to their tastes and preferences in a boiling broth on the dining table. The entire process is similar to the co-creation service experience, wherein companies allow customers to co-create their own experience (Prahalad and Ramaswamy, 2004). It is similar to customized offerings where actors invite other actors to participate in the production of service offerings (Mathis et al., 2016). We collected data from international travelers who dined at hotpot restaurants during their stay in China.

Accordingly, we developed a structured questionnaire divided into three parts. In the first part, we explained the study purpose and asked respondents to recall their recent experiences at a hotpot restaurant and answer questions on a given scale to describe their experience and satisfaction. We assured them of the confidentiality of the information provided. In the second part, we collected the respondents’ demographic data and their country of origin. The third part comprised items that assessed the variables of interest. The current research conducted an online questionnaire, employing a structured and non-probabilistic convenience sample and cross-sectional survey. We conducted our survey questionnaire on Sojump platform. We shared the link among online group chats of foreigners living in China with the help of group chat administrators as it is the fastest way to access a large sample group in a short period of time. We forwarded the questionnaire along with a message stating that only foreigners who have dined at a Chinese hotpot restaurant within the last 3 months are eligible to fill the survey. Consequently, we distributed 600 self-administered questionnaires and received 521 responses within a month. Of these responses, 68 were excluded from the analysis because they contained unengaged responses and missing information. Therefore, the final sample comprised 453 foreign respondents (response rate of 75.5%) from 61 countries across six continents.

Our sample included 291 men (64.2%) and 158 women (34.9%). The majority of respondents (81%) were aged between 26 and 55 years. Additionally, 96.5% had at least a bachelor’s degree, and 66.7% were employed (public and private sector) or self-employed (businesspersons). Table 1 reports the demographic details of the respondents.

**Table 1:** Demographics of respondents.

Items	Frequency *N* = 453	Percentage
*Gender*		
Male	291	64.2
Female	158	34.9
Other	4	0.9
*Age*		
18–25Y	80	17.7
26–35Y	257	56.7
36–45Y	93	20.5
46–55Y	17	3.8
55 years or above	6	1.3
*Education*		
High school or less	13	2.9
Bachelor	201	44.4
Master’s	176	38.9
PhD	60	13.2
Other	3	0.7
*Profession*		
Student	119	26.3
Employee (Private sector)	193	42.6
Employee (Public sector)	57	12.6
Businessman/Businesswoman	52	11.5
Unemployed	7	1.5
Other	25	5.5
*Continent*		
Africa	129	28.5
Asia	64	14.1
Australia	11	2.4
Europe	131	28.9
North America	106	23.4
South America	12	2.7

*Y, years*.

### Data Analysis

The present research used IBM SPSS Statistics version 26.0 to analyze the demographics and the common method bias. We assessed our measurement and structural model with partial least square structural equation modeling (PLS-SEM) on SmartPLS version 3.3.3. In recent years, PLS has become a very popular and powerful alternative to covariance explanation methods, such as AMOS and LISREL (Wasko and Faraj, 2005). PLS is better suited for explaining complex relationships, and it synchronously estimates the measurement and the structural model. The data were analyzed in two stages: In the first stage, we tested data reliability and validity, and in the second stage, we tested the hypotheses.

## Results

The data were analyzed in two stages: In the first stage, we tested data reliability and validity, and in the second stage, we tested the hypotheses.

### Common Method Bias

Common method bias is a crucial issue in behavioral research. We tested it using Harman’s single-factor approach. The variance extracted by a single factor was 25.099%, which is less than 50%, indicating no common method bias in this study.

### Assessment of Measurement Model

Ringle et al. (2015) stated that the measurement model must establish reliability and validity. In the reliability analysis, the alpha and composite reliability were higher than 0.7. For convergent validity, the standardized factor loadings were greater than 0.5. Furthermore, the average variance extracted (AVE) values were greater than 0.5 and met the threshold level. Table 2 presents details on composite reliability (CR), alpha, AVE, and outer loading.

**Table 2:** Alpha, composite reliability, and average variance extracted values.

Constructs	Items	Loadings	Alpha	CR	AVE
HE	HE_1	0.800	**0.776**	**0.868**	**0.687**
	HE_2	0.851			
	HE_3	0.836			
CoE	CoE_1	0.820	**0.85**	**0.892**	**0.624**
	CoE_2	0.766			
	CoE_3	0.812			
	CoE_4	0.797			
	CoE_5	0.750			
SE	SE_1	0.781	**0.866**	**0.898**	**0.595**
	SE_2	0.801			
	SE_3	0.728			
	SE_4	0.792			
	SE_5	0.757			
	SE_6	0.769			
EE	EE_1	0.733	**0.848**	**0.890**	**0.619**
	EE_2	0.800			
	EE_3	0.772			
	EE_4	0.831			
	EE_5	0.792			
OCE	OCE_1	0.831	**0.829**	**0.886**	**0.66**
	OCE_2	0.814			
	OCE_3	0.806			
	OCE_4	0.798			
BP	BP_1	0.884	**0.936**	**0.951**	**0.797**
	BP_2	0.896			
	BP_3	0.903			
	BP_4	0.884			
	BP_5	0.895			
BT	BT_1	0.886	**0.933**	**0.949**	**0.789**
	BT_2	0.897			
	BT_3	0.873			
	BT_4	0.892			
	BT_5	0.895			
BPI	BPI_1	0.902	**0.926**	**0.948**	**0.819**
	BPI_2	0.900			
	BPI_3	0.912			
	BPI_4	0.905			
PBR	PBR_1	0.911	**0.891**	**0.932**	**0.821**
	PBR_2	0.904			
	PBR_3	0.903			
OBR	OBR_1	0.923	**0.905**	**0.941**	**0.841**
	OBR_2	0.914			
	OBR_3	0.913			

*Alpha, Cronbach Alpha, CR, composite reliability and AVE, average variance extracted*.

For discriminant validity, we applied two methods, namely, the Fornell Larcker criterion and the heterotrait–monotrait (HTMT) ratio of correlations. In the Fornell and Larcker method, the square root of each latent variable’s AVE was greater than the correlation of its coefficient, which indicates discriminant validity in our study (Fornell and Larcker, 1981). Henseler et al. (2015) stated that the HTMT values must be lower than 0.85, which was the case in our study, indicating discriminant validity (see Table 3). We further examined the variance inflation factor (VIF), and the VIF values did not exceed 5; therefore, multicollinearity was acceptable in this study.

**Table 3:** Fornell and Larcker criterion and heterotrait–monotrait ratio.

Construct	Mean	SD	1	2	3	4	5	6	7	8	9	10
1. BP	3.25	1.18	**0.893**	0.634	0.575	0.11	0.117	0.113	0.622	0.09	0.616	0.165
2. BPI	3.25	1.19	0.591	**0.905**	0.574	0.113	0.139	0.153	0.587	0.111	0.637	0.16
3. BT	3.29	1.15	0.538	0.534	**0.888**	0.1	0.142	0.088	0.565	0.101	0.6	0.041
4. CoE	3.77	0.80	0.1	0.104	0.088	**0.79**	0.141	0.14	0.145	0.103	0.128	0.126
5. EE	3.75	0.80	0.113	0.129	0.128	−0.113	**0.786**	0.142	0.118	0.068	0.163	0.104
6. HE	3.75	0.83	0.1	0.131	0.078	−0.1	−0.12	**0.829**	0.128	0.061	0.149	0.067
7. OBR	3.23	1.19	0.573	0.538	0.519	0.127	0.109	0.109	**0.917**	0.128	0.621	0.119
8. OCE	3.71	0.84	0.081	0.099	0.091	−0.084	−0.038	−0.046	0.114	**0.812**	0.17	0.067
9. PBR	3.25	1.19	0.563	0.578	0.548	0.113	0.148	0.127	0.558	0.147	**0.906**	0.17
10. SE	3.76	0.78	0.16	0.152	0.009	−0.11	−0.071	−0.055	0.11	−0.052	0.157	**0.772**

*Values on the diagonal (bold) represent the square root of the average variance extracted, while the off-diagonals are correlations; SD, standard deviation*.

### Assessment of Structural Model

To conduct hypothesis testing, the statistical bootstrap technique was applied with the recommended sample size of 5,000 using SmartPLS software version 3.3.3. (Ringle et al., 2015).

H1’s findings revealed that CCE dimensions, namely, HE (*β* = 0.96, 0.105, and 0.071), CoE (*β* = 0.075, 0.099, and 0.091), SE (*β* = 0.11, 0.133, and 0.068), EE (BPI: *β* = 0.078 and PBR: *β* = 0.107), and OCCE (*β* = 0.06, 0.114, and 0.075) positively impact the BPI, PBR, and OBR dimensions of BE. However, the relationship between EE and OBR is insignificant (*β* = 0.057).

Furthermore, CCE dimensions, including HE (*β* = 0.119), CoE (*β* = 0.134), EE (*β* = 0.165), and OCCE (*β* = 0.117) directly contribute to enhancing BT, whereas SE (*β* = 0.048) was found to be non-significant. The analysis of H3 revealed that CCE dimensions, namely, HE (*β* = 0.153), CoE (*β* = 0.167), SE (*β* = 0.204), EE (*β* = 0.169), and OCCE (*β* = 0.119) directly increase BP.

Findings for H4 highlighted that BT strongly impacts BE dimensions, namely, BPI (*β* = 0.298), PBR (*β* = 0.333), and OBR (*β* = 0.287). Additionally, results revealed that BP has a positive and significant impact on the BPI (*β* = 0,382), PBR (*β* = 0.321), and OBR (*β* = 0.38) dimensions of BE. Table 4 illustrates the direct effect.

**Table 4:** Hypotheses testing direct effect.

Hypothesis	Direct relationships	*β*	Std. Error
H1a1	HE→BPI	0.096*	0.038
H1a2	HE→PBR	0.105**	0.031
H1a3	HE→OBR	0.071*	0.033
H1b1	CoE→BPI	0.075*	0.034
H1b2	CoE→PBR	0.099**	0.030
H1b3	CoE→OBR	0.091*	0.042
H1c1	SE→BPI	0.11**	0.038
H1c2	SE→PBR	0.133***	0.035
H1c3	SE→OBR	0.068*	0.033
H1d1	EE→BPI	0.078*	0.030
H1d2	EE→PBR	0.107**	0.035
H1d3	EE→OBR	0.057^NS^	0.031
H1e1	OCCE→BPI	0.06*	0.028
H1e2	OCCE→PBR	0.114**	0.040
H1e3	OCCE→OBR	0.075*	0.034
H2a	HE→BT	0.119*	0.051
H2b	CoE→BT	0.134*	0.059
H2c	SE→BT	0.048^NS^	0.054
H2d	EE→BT	0.165**	0.053
H2e	OCCE→BT	0.117*	0.050
H3a	HE→BP	0.153**	0.048
H3b	CoE→BP	0.167**	0.049
H3c	SE→BP	0.204***	0.047
H3d	EE→BP	0.169**	0.051
H3e	OCCE→BP	0.119*	0.050
H4a	BT→BPI	0.298***	0.043
H4b	BT→PBR	0.333***	0.041
H4c	BT→OBR	0.287***	0.041
H6a	BP→BPI	0.382***	0.045
H6b	BP→PBR	0.321***	0.045
H6c	BP→OBR	0.38***	0.045

*NS, not significant, std, standard, and *β*, Beta*.

* *p* *< 0.05;*

** *p* *< 0.01;*

*** *p < 0.001*.

The mediation effect results demonstrated that BT mediates the relationship between the CCE dimensions, including HE (*β* = 0.035, 0.04, and 0.034), CoE (*β* = 0.04, 0.034, and 0.038), EE (*β* = 0.049, 0.055, and 0.047) and OCCE (*β* = 0.035, 0.039, and 0.034), and BE dimensions of BPI, PBR, and OBR. However, BT does not mediate the relationship between SE and BE dimensions (H5).

**Figure 2:**
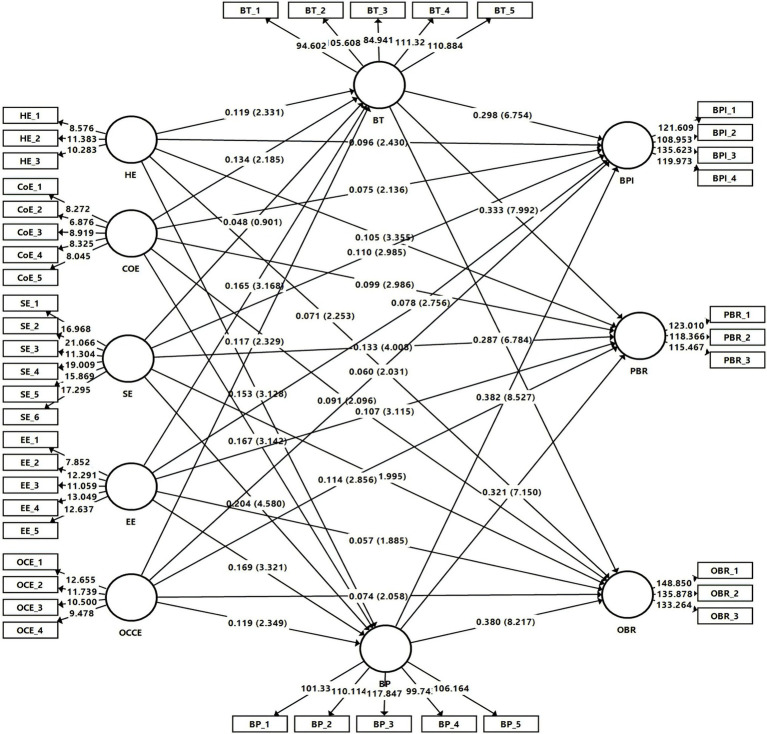
Assessment of Structural Model.

Furthermore, we found that BP mediates the relationship between the CCE dimensions, namely, HE (*β* = 0.059, 0.049, and 0.058), CoE (*β* = 0.064, 0.054, and 0.064), SE (*β* = 0.076, 0.066, and 0.078), EE (*β* = 0.065, 0.054, and 0.064) and OCCE (*β* = 0.046, 0.039, and 0.046), and BE dimensions, namely, BPI, PBR, and OBR (H7; see Table 5). The structural model is given in Figure 2.

**Table 5:** Hypotheses testing indirect effect.

Hypothesis	Indirect relationships	*β*	Std. Error
H5a1	HE→BT→BPI	0.035*	0.017
H5a2	HE→BT→PBR	0.04*	0.019
H5a3	HE→BT→OBR	0.034*	0.017
H5b1	CoE→BT→BPI	0.04*	0.02
H5b2	CoE→BT→PBR	0.045*	0.021
H5b3	CoE→BT→OBR	0.038*	0.019
H5c1	SE→BT→BPI	0.014^NS^	0.017
H5c2	SE→BT→PBR	0.016^NS^	0.019
H5c3	SE→BT→OBR	0.014^NS^	0.017
H5d1	EE→BT→BPI	0.049**	0.018
H5d2	EE→BT→PBR	0.055**	0.02
H5d3	EE→BT→OBR	0.047**	0.018
H5e1	OCCE→BT→BPI	0.035*	0.016
H5e2	OCCE→BT→PBR	0.039*	0.018
H5e3	OCCE→BT→OBR	0.034*	0.016
H7a1	HE→BP→BPI	0.059**	0.02
H7a2	HE→BP→PBR	0.049**	0.018
H7a3	HE→BP→OBR	0.058**	0.02
H7b1	CoE→BP→BPI	0.064**	0.022
H7b2	CoE→BP→PBR	0.054**	0.019
H7b3	CoE→BP→OBR	0.064**	0.021
H7c1	SE→BP→BPI	0.078***	0.022
H7c2	SE→BP→PBR	0.066**	0.019
H7c3	SE→BP→OBR	0.078***	0.022
H7d1	EE→BP→BPI	0.065**	0.022
H7d2	EE→BP→PBR	0.054**	0.019
H7d3	EE→BP→OBR	0.064**	0.022
H7e1	OCCE→BP→BPI	0.046*	0.021
H7e2	OCCE→BP→PBR	0.039*	0.018
H7e3	OCCE→BP→OBR	0.046*	0.021

*NS, not significant, std, standard, and *β*, Beta*.

* *p* *< 0.05;*

** *p* *< 0.01;*

*** *p < 0.001*.

*R*-squared is the proportion of an endogenous construct’s variance explained by its predictor constructs. According to Hair et al. (2011), 0.25, 0.50, and 0.75 denote small, medium, and large effects. Table 6 represents the adjusted R^2^ values.

**Table 6:** Adjusted *R*^2^ value.

Constructs	*R* ^2Adj^
BP	0.083
BPI	0.429
BT	0.043
OBR	0.399
PBR	0.433

*R^2Adj^ = adjusted R square value*.

## Discussion

Currently, the world is rapidly developing and globalization is influencing all countries. Accordingly, several tourism and hospitality markets around the world are trying to figure out ways to improve their services and increase customer satisfaction. This study focuses on value co-creation and investigates the impact of international tourists’ CCE on BE, BT, and BP.

The first hypothesis’s findings revealed that CCE dimensions, namely, HE, SE, and OCCE positively impact the BPI, PBR, and OBR dimensions of BE. A plausible reason is that customers who participated in the value co-creation activity feel less deprived and more fulfilled (Navarro et al., 2015); therefore, they will actively buy the brand, recommend it to others and defend it from any form of criticism. Customers are more likely to show behavioral support to a brand that enriches their social lives, entertains them, and provides new knowledge and information. The findings further revealed that the relationship between EE and OBR is insignificant. This results in the fact that EE does not sufficiently engage international travelers in evangelistic behaviors. International travelers actively purchase the brand and recommend it to others; however, spreading negative opinions about other restaurant brands is perceived as a burden to them.

Furthermore, CCE dimensions, including HE, CoE, EE, and OCCE directly contribute to enhancing BT; however, SE was found to be non-significant. This may be because international travelers seek to connect with like-minded people and gain better self-esteem through co-creation activity; however, they do not necessarily place their confidence in the brand and rely on it. The analysis of the third hypothesis revealed that CCE dimensions directly increase BP, thus enriching extant hospitality and tourism literature. These findings are reasonable, as engaged customers are key to a brand’s success. Findings suggest that customers who participate in the co-creation process develop a high level of enthusiasm and desire and are emotionally connected to the brand. Passion is achieved by providing customers with real value through every aspect of the customer–brand relationship.

Findings for the fourth hypothesis highlighted that BT strongly impacts BE dimensions, namely, BPI, PBR, and OBR, which is in line with extant literature (Badrinarayanan and Becerra, 2013; Doss, 2014; Riorini and Widayat, 2015). These findings suggest that when customers trust a brand, they actively purchase it and engage many other customers. Customers’ trust in a brand reflects a positive feeling and the willingness to be loyal to it, therefore engendering brand evangelistic behaviors. Additionally, The mediation effect results of the fifth hypothesis demonstrated that BT mediates the relationship between the CCE dimensions, including HE, CoE, EE, and OCCE, and BE dimensions of BPI, PBR, and OBR. However, BT does not mediate the relationship between SE and BE dimensions. A reasonable explanation is that social relationships do not lead to brand trust. International travelers do not necessarily rely on the brand, which, in turn, affects their behavior.

Moreover, results for the sixth hypothesis revealed that BP has a positive and significant impact on the BPI, PBR, and OBR dimensions of BE, consistent with Matzler et al. (2007), enriching extant BE literature. Passion is deemed the core of the emotional connection between customers and a brand. Passionate customers are more enthusiastic and are considered profitable advocates of a brand. Their behavior goes beyond purchasing a specific brand to recommend it and denigrate rival brands that present a threat. Furthermore, we found that BP mediates the relationship between the CCE dimensions, namely, HE, CoE, SE, EE, and OCCE, and BE dimensions, namely, BPI, PBR, and OBR. These findings are reasonable as when customers who participated in the co-creation activity had a positive experience, they become passionate about the brand, and therefore, engage in evangelistic behaviors (Doss, 2014). These findings are consistent with Pansari and Kumar's (2017) theory of engagement.

### Theoretical Implications

The current study contributes to the branding, hospitality, and tourism literature in multiple ways. First, Im and Qu (2017) stressed that most studies on co-creation are scenario-based. This study examined the proposed conceptual model in a realistic co-creation setting (i.e., hotpot restaurants) that explains how international tourists’ CCE can enhance BT, increase BP, and stimulate brand evangelistic behaviors, offering theoretical grounds for future research on CCE and BE.

Second, extant literature suggested that a positive customer experience is necessary to achieve performance and relational benefits (Hussain et al., 2020). However, such experience must be scrutinized at a dimensional level to understand how each dimension affects consumer behavior. To the best of our knowledge, the current study is the first attempt to reveal how the multidimensional concept of CCE affects the multidimensional concept of BE. Our research is also the first to investigate CCE and branding relationships, including BT and BP.

Third, the post-hoc analysis investigating BT and BP’s mediating role between CCE and BE is a valuable contribution. The findings highlighted that BT has a positive mediation effect on CCE dimensions, including HE, CoE, EE, and OCCE, and BE dimensions, namely, BPI, PBR, and OBR, whereas the SE mediation effect is non-significant. Additionally, BP was found to mediate all dimensions of CCE and BE.

Fourth, research on CCE is limited in the hospitality literature with respect to international tourists’ perspectives. Previous research has investigated the CCE of local diners (i.e., Hussain et al., 2020). The current study investigates international tourists’ CCE and its impact on BE.

Fifth, BE has been investigated in banking service (Riorini and Widayat, 2015), the video game industry (Marticotte et al., 2016), and social media use (Harrigan et al., 2020). The current research introduces the concept of BE in the hospitality industry; specifically, it investigates how the dining experience of international tourists instigates brand evangelistic behaviors.

Finally, BE has been a hot topic in marketing and branding literature (Harrigan et al., 2020). The literature has treated BE as a higher-order construct (e.g., Matzler et al., 2007) or a construct with two dimensions (e.g., Harrigan et al., 2020). This study considered BE as a construct with three dimensions: BPI, PBR, and OBR, further clarifying the BE phenomenon.

### Managerial Implications

As we evolve rapidly to consider CCE as the basis of value, the interaction between the company and the customer becomes the locus of value co-creation (Prahalad and Ramaswamy, 2004). The current study provides valuable insight for an in-depth understanding of foreign tourists with respect to restaurants engaged in the profitable Chinese marketplace.

The findings suggest that firms should engage customers in the co-creation of value where the interaction between customer and employee is crucial to increase customers’ BT, BP, and brand evangelistic behaviors. To stimulate optimized customer experience for brand success, restaurant managers can focus on the various aspects of value co-creation (i.e., HE, CoE, SE, EE, and OCCE). For instance, they can allow tourists to co-create a service experience that suits their context, make the brand engaging and meaningful, and create a better customer experience. Managers can also provide an innovative experience environment for new co-creation experiences (Prahalad and Ramaswamy, 2004) and provide customer support to help customers co-create. Optimizing CCE will cause customers to show their passion for the brand and rely on it. Consequently, customers will develop brand evangelistic behaviors, such as PBR, which help attract additional customers who may otherwise not be attracted *via* conventional marketing channels.

Pansari and Kumar's (2017) theory of engagement states that the consumer–brand relationship is neither static nor instant. Therefore, restaurant brand managers must stimulate a positive customer experience, which will eventually enhance BP and lead to brand evangelistic behaviors. A passionate customer is an asset to a brand. Brand managers can instill BP by implementing and providing platforms and tools for a pleasurable CCE (HE), as customers participating in the value co-creation activity seek fun, pleasure, and entertainment.

Additionally, restaurant brand managers can offer customers the opportunity to learn about service, products, and technologies (CoE) and provide a suitable platform for them to connect with like-minded people (SE). Customers involved in the co-creation process give suggestions and feedback, which reduce risks associated with offering undesirable products or services. Further, managers can provide compensation commensurate with the effort made during the co-creation process (pragmatic and economic benefit). For instance, they could formulate a reward system (company swag) and focus on sophisticated reward programs.

### Limitations and Recommendations

The current study has several limitations. First, we used a structured questionnaire to assess international tourists’ participation. Given that it may limit tourists’ expression, future research may employ a mixed-methods approach. Second, we focused on co-creation in a restaurant setting; future research could specifically focus on co-creation activities in tourism services and online shopping. Third, we did not consider the control variables’ role in this research. Therefore, future research may include control variables, such as tourists’ gender, age, and the frequency of dining at a hotpot restaurant. Fourth, we associated BT and BP to understand the relationship between CCE and BE. Researchers may use other variables, such as customer perceived value, brand love, and affective commitment to better understand the relationship.

## Data Availability Statement

The raw data supporting the conclusions of this article will be made available by the authors, without undue reservation.

## Ethics Statement

The studies involving human participants were reviewed and approved by East China University of Science and Technology. Written informed consent to participate in this study was provided by the participants’ legal guardian/next of kin.

## Author Contributions

GN provided writing, editing, data collection, initial analysis, methodology, and conceptualization. FJ and KH provided review and critical advices. SJ and MR worked on the result. All authors contributed to the article and approved the submitted version.

## Conflict of Interest

The authors declare that the research was conducted in the absence of any commercial or financial relationships that could be construed as a potential conflict of interest.

## Publisher’s Note

All claims expressed in this article are solely those of the authors and do not necessarily represent those of their affiliated organizations, or those of the publisher, the editors and the reviewers. Any product that may be evaluated in this article, or claim that may be made by its manufacturer, is not guaranteed or endorsed by the publisher.

## Abbreviations

CCE: Co-creation experience
HE: Hedonic experience
SE: Social experience
EE: Economic experience
CoE: Cognitive experience
OCCE: Overall co-creation experience
BE: Brand evangelism
BPI: Brand purchase intention
PBR: Positive brand referrals
OBR: Oppositional brand referrals
BT: Brand trust
BP: Brand passion
